# Muscle-fiber array inspired, multiple-mode, pneumatic artificial muscles through planar design and one-step rolling fabrication

**DOI:** 10.1093/nsr/nwab048

**Published:** 2021-03-24

**Authors:** Jiang Zou, Miao Feng, Ningyuan Ding, Peinan Yan, Haipeng Xu, Dezhi Yang, Nicholas X Fang, Guoying Gu, Xiangyang Zhu

**Affiliations:** State Key Laboratory of Mechanical System and Vibration, Shanghai Jiao Tong University, Shanghai 200240, China; Robotics Institute, School of Mechanical Engineering, Shanghai Jiao Tong University, Shanghai 200240, China; State Key Laboratory of Mechanical System and Vibration, Shanghai Jiao Tong University, Shanghai 200240, China; Robotics Institute, School of Mechanical Engineering, Shanghai Jiao Tong University, Shanghai 200240, China; State Key Laboratory of Mechanical System and Vibration, Shanghai Jiao Tong University, Shanghai 200240, China; Robotics Institute, School of Mechanical Engineering, Shanghai Jiao Tong University, Shanghai 200240, China; State Key Laboratory of Mechanical System and Vibration, Shanghai Jiao Tong University, Shanghai 200240, China; Robotics Institute, School of Mechanical Engineering, Shanghai Jiao Tong University, Shanghai 200240, China; State Key Laboratory of Mechanical System and Vibration, Shanghai Jiao Tong University, Shanghai 200240, China; Robotics Institute, School of Mechanical Engineering, Shanghai Jiao Tong University, Shanghai 200240, China; State Key Laboratory of Mechanical System and Vibration, Shanghai Jiao Tong University, Shanghai 200240, China; Robotics Institute, School of Mechanical Engineering, Shanghai Jiao Tong University, Shanghai 200240, China; Department of Mechanical Engineering, Massachusetts Institute of Technology, Cambridge, MA 02139, USA; State Key Laboratory of Mechanical System and Vibration, Shanghai Jiao Tong University, Shanghai 200240, China; Robotics Institute, School of Mechanical Engineering, Shanghai Jiao Tong University, Shanghai 200240, China; State Key Laboratory of Mechanical System and Vibration, Shanghai Jiao Tong University, Shanghai 200240, China; Robotics Institute, School of Mechanical Engineering, Shanghai Jiao Tong University, Shanghai 200240, China

**Keywords:** soft robotics, pneumatic artificial muscles, multiple-mode actuations, bio-inspired design

## Abstract

Advances in development of artificial muscles have enabled creation of soft robots with biological dexterity and self-adaption in unstructured environments; however, production of scalable artificial muscles with multiple-mode actuations remains elusive. Inspired by muscle-fiber arrays in muscular hydrostats, we present a class of versatile artificial muscles called MAIPAMs (muscle-fiber array inspired pneumatic artificial muscles), capable of multiple-mode actuations (such as parallel elongation-bending-spiraling actuations, 10 parallel bending actuations and cascaded elongation-bending-spiraling actuations). Our MAIPAMs consist of active 3D elastomer-balloon arrays reinforced by a passive elastomer membrane, achieved through a planar design and one-step rolling fabrication approach. We introduce prototypical designs for the MAIPAMs and demonstrate their muscle-mimic structures and versatility, as well as their scalable ability to integrate flexible but non-stretchable layers for contraction and twisting actuation modes and compliant electrodes for self-sensing. We further demonstrate that this class of artificial muscles shows potential for versatile robotic applications, such as carrying a camera for recording videos, gripping or manipulating objects, and climbing a pipe-line.

## Teaser

We demonstrate the first drone-based entanglement distribution under multi-weather conditions, which exhibits the third possible experimental strategy for quantum communication, in addition to fiber and satellite.

## INTRODUCTION

Soft robots, mimicking the functions and movements of biological organisms, such as locomotion and manipulation, have recently attracted great interest within the field of robotics [1–3]. Differing from traditional robots made of rigid materials, soft robots are mainly composed of muscle-like materials for the actuation mechanism (known as artificial muscles) [4–7]. Shape memory polymers [8,9], stimuli-responsive polymers/elastomers [10–13] and pneumatic actuators [14–17] are commonly used artificial muscles.

Among these, pneumatic actuators have been widely reported for use in soft robots [18,19] because of their large deformation, high output force and easy control. During the past two decades, different pneumatic actuators with various actuation modes have been developed (see refs [6,14,20] for overviews of different pneumatic actuators). McKibben actuators [21,22] are an early example of pneumatic actuators, which mainly consist of a non-stretchable bladder wrapped in inextensible fibers. With the development of soft materials (such as silicone rubber), fiber-reinforced actuators [23,24] and PneuNet actuators [25,26] have become more popular because of their compliance and safety. By designing a special fiber angle (for McKibben and fiber-reinforced actuators) or chamber angle (for PneuNet actuators), different actuation modes can be generated, such as bending, elongation, contraction or twisting. However, existing pneumatic actuators are generally limited to a single-mode actuation [27], hindered by the lack of easy design and fabrication approaches for multiple-mode actuations [28,29].

**Figure 1.**
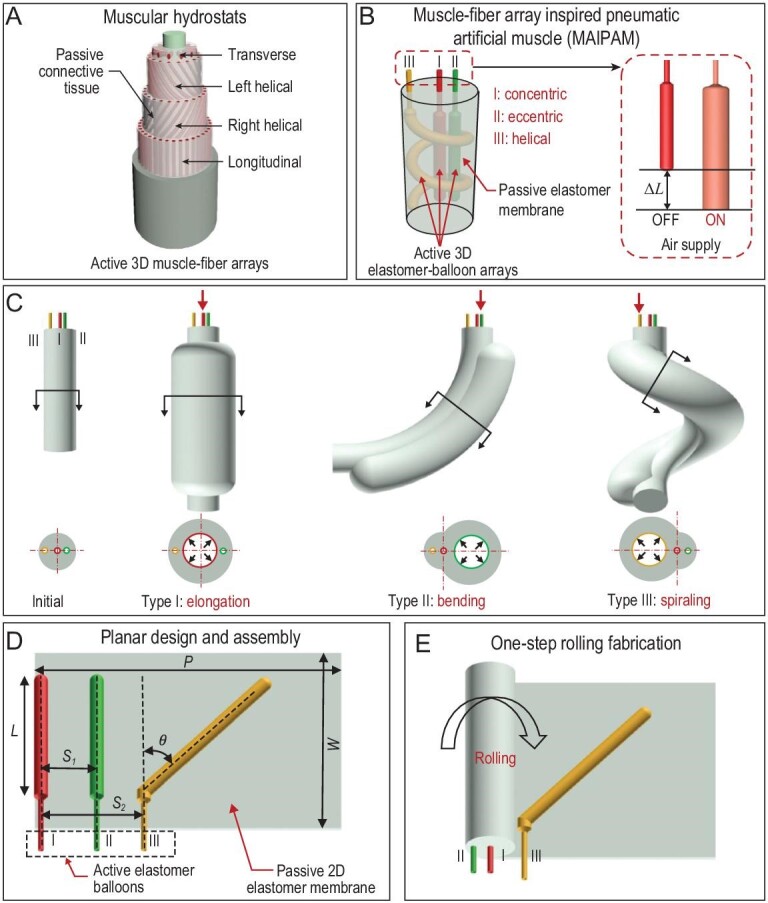
Design and fabrication principle of the MAIPAMs. (A) Schematic of the skeleton-free muscular hydrostats. Muscular hydrostats mainly consist of active three-dimensional (3D) muscle-fiber arrays (including transverse, left-helical, right-helical and longitudinal muscle fibers) reinforced by passive connective tissues, enabling multiple-mode actuations and versatile manipulation by selectively actuating the muscle-fiber arrays. (B) The morphology of the designed MAIPAMs, consisting of active 3D elastomer-balloon arrays (cylinder latex elastomer balloon with an outer diameter of 5 mm and a wall thickness of 0.3 mm) reinforced by a passive 2D elastomer membrane (3M VHB 4910 with inherent strong adhesive, a thickness of 1 mm). The arrays contain three kinds of active elastomer balloons: concentric (type I), eccentric (type II) and helical (type III) active elastomer balloons. (C) Multiple-mode actuations of the MAIPAMs. Upon a supplied pressure, the elongation of active elastomer balloons can be converted into elongation (type I balloon), bending (type II balloon) and spiraling (type III balloon), respectively. (D) Schematic of the planar design and assembly of the MAIPAMs (Fig. S2 and Movie S1): (i) determine the design parameters of active elastomer balloons (including the length *L* of the active elastomer balloon, the distance *S* between the active elastomer balloon and the edge of the passive elastomer membrane, and the oblique angle $\theta $); (ii) assemble the active elastomer balloons in a passive 2D elastomer membrane. (E) Working principle of the one-step fabrication approach for rolling the passive 2D elastomer membrane into a 3D MAIPAM (Movie S1).

In biological organisms (such as elephant trunk, human tongues and octopus tentacles), skeleton-free muscular hydrostats consisting of active 3D muscle-fiber arrays (including transverse, left-helical, right-helical and longitudinal muscle fibers, Fig. 1A) reinforced by passive connective tissues, demonstrate remarkable ability for multiple-mode actuations and versatility [30,31]. Inspired by these active 3D muscle-fiber arrays, recently, some pneumatic elastomer actuators have been developed to generate different actuation modes through reconfigurable fiber-based limiting laminae [28] or multi-material 3D printed fiber arrays [29]. However, these still fail to achieve multiple-mode actuations with one pneumatic elastomer actuator because these fibers work only as passive limiting structures, unlike the active muscle fibers in muscular hydrostats. Therefore, achieving multiple-mode actuations for pneumatic elastomer actuators remains a challenge in the field of soft robotics.

Here, we present a class of active 3D muscle-fiber array inspired pneumatic artificial muscles (termed muscle-fiber array inspired pneumatic artificial muscles (MAIPAMs), Fig. 1B) that demonstrate the ability of producing multiple-mode actuations similar to muscular hydrostats. The MAIPAMs consist of active 3D elastomer-balloon arrays reinforced by a passive elastomer membrane. The active elastomer balloon can generate an elongation under compressed air while the passive elastomer membrane can transform the elongation into multiple-mode actuations, including elongation, bending and spiraling (Fig. 1C). To design and fabricate the active 3D elastomer-balloon arrays of the MAIPAMs, we propose a planar design and one-step rolling fabrication approach (Fig. 1D and E, Movie S1 in the online supplementary file). By design of the position and the number of elastomer balloons, MAIPAMs can achieve parallel multiple-mode actuations (such as the parallel elongation-bending-spiraling seen in Movie S2; parallel 10 bending actuations for omnidirectionally recording videos in a confined space seen in Movie S3) and cascaded multiple-mode actuations (such as the cascaded elongation-bending-spiraling seen in Movie S4 and its application for gripping seen in Movie S5). The planar design and one-step rolling fabrication approach also enables the MAIPAMs to conveniently integrate limiting layers for contraction and twisting actuation modes and compliant electrodes for a self-sensing ability. We demonstrate that the contracted MAIPAMs can be used to actuate a robotic arm (Movie S6) and the MAIPAMs with a self-sensing module can be used for closed-loop position control and object manipulation (Movie S7). We further demonstrate that the multiple-mode MAIPAMs can be used to build an untethered soft pipe-climbing robot, capable of stable climbing in a pipe-line with a diameter of 55 mm (Movie S8).

## RESULTS

### Design and fabrication principle of the MAIPAMs

Inspired by the structure of active 3D muscle-fiber arrays in muscular hydrostats (Fig. 1A), we designed a MAIPAM (Fig. 1B) composed of active 3D elastomer-balloon arrays (cylinder latex elastomer balloon with a outer diameter of 5 mm and a wall thickness of 0.3 mm, Fig. S1A) reinforced by a passive elastomer membrane (3M VHB 4910 with inherent strong adhesives, a thickness of 1 mm, Fig. S1B). The active 3D elastomer-balloon arrays consist of three types of elastomer balloons: concentric elastomer balloon (type I, concentric with the long axis), eccentric elastomer balloon (type II, off-centered but parallel with the long axis) and helical elastomer balloon (type III, forming a helical shape). Upon supplied pressure, each active elastomer balloon can generate an elongation while the passive elastomer membrane converts the elongation into multiple-mode actuations (Fig. 1C). For instance, the elongation of the type I balloon leads to a uniform deformation of the MAIPAMs because of their symmetric structure, generating an elongation mode. The elongation of the type II balloon generates a non-uniform deformation of the MAIPAMs because of their asymmetric structure, achieving a bending mode. The elongation of the type III balloon produces a coupled bending and twisting deformation of the MAIPAMs because of their helical shape, forming a spiraling mode.

To fabricate the active 3D elastomer-balloon arrays for the MAIPAMs, we developed a planar design and rolling approach (Fig. S2 and Movie S1), including the following steps: i) determine the design parameters of the active elastomer balloons (i.e. the length *L* of the active elastomer balloon, the distance *S* between the active elastomer balloon and the edge of the passive elastomer membrane, and the oblique angle $\theta $; see supplementary text, Table S1 and Fig. S3 for the relationship between the design parameters and the 3D structure of the MAIPAMs); ii) assemble the active elastomer balloons in a passive 2D elastomer membrane (Fig. 1D); iii) roll the passive 2D elastomer membrane with the active elastomer-balloon arrays into a 3D MAIPAM (Fig. 1E).

### Influences of design parameters on the actuation modes of the MAIPAMs

The actuation modes of the MAIPAMs depend mainly on three parameters (*L*, *S*, $\theta $) of the active elastomer balloon in the passive 2D elastomer membrane. We firstly investigated how the set of parameters (*L*, *S*, $\theta $) influenced the actuation mode and characterized performance of the MAIPAMs with one active elastomer balloon. To describe the relationship between the (*L*, *S*, $\theta $) and the 3D structure of the MAIPAMs, we developed a geometric model (see supplementary text). Using that geometric model, we obtained the position of the active elastomer balloon in the MAIPAMs (Table S1). It should be noted that we kept the geometric parameters of the passive elastomer membrane constant (i.e. length *P* = 300 mm, thickness *t* = 1 mm. Selection of width *W* needs to ensure that the distances between two ends of the active elastomer balloon and edges of the passive elastomer membrane are >10 mm).

#### Elongation

**Figure 2.**
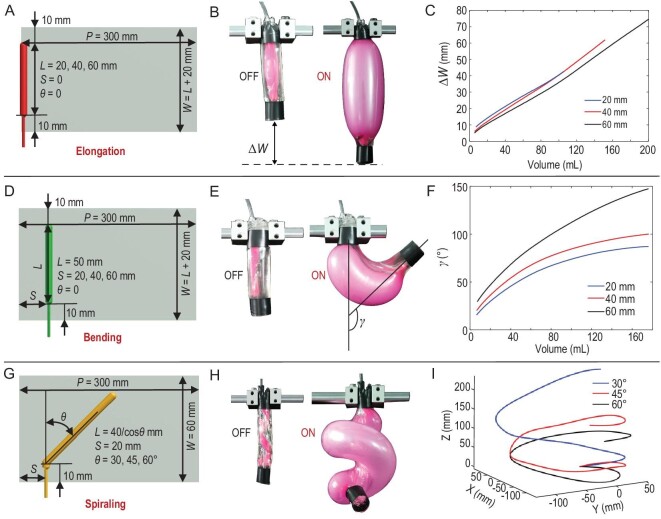
Characterization of the design parameters on the actuation modes of the MAIPAMs. (A) Schematic of the 2D-based design pattern of the elongated MAIPAMs. An active elastomer balloon (type I) is assembled at position (*L* > 0, *S = 0*, $\theta $*= 0*) in the passive 2D elastomer membrane. (B) The working principle of the elongated MAIPAMs: upon supplied volume *V* of the compressed air, the MAIPAMs generate an elongated displacement $\Delta W$. (C) $\Delta W$ is plotted as a function of *V* when *L* equals 20, 40 and 60 mm, respectively. (D) Schematic of the 2D-based design pattern of the bending MAIPAMs. An active elastomer balloon (type II) is assembled at position (*L* = 50 mm, *S > 0*, $\theta $*= 0*) in the passive 2D elastomer membrane. (E) The working principle of the bending MAIPAMs: upon supplied volume *V* of the compressed air, the MAIPAMs generate a bending angle $\gamma $. (F) $\gamma $ is plotted as a function of *V* when *S* equals 20, 40 and 60 mm, respectively. (G) Schematic of the 2D-based design pattern of the spiraling MAIPAMs. An active elastomer balloon (type III) is assembled at position (*L *= 40/cos$\theta $ mm, *S =* 20* *mm, $\theta $*>0*) in the passive 2D elastomer membrane. (H) The working principle of the spiraling MAIPAMs: upon supplied volume *V* of the compressed air, they form helical shapes. (I) The helical shapes of the spiraling MAIPAMs with different $\theta $ when *V* equals 120 mL with a pressure of 46.5 kPa.

To form a concentric elastomer balloon (type I) for the elongation actuation, both *S* and $\theta $ were set as zero, and *L* was the primary design parameter. Therefore, we designed the elongated MAIPAMs by assembling an active elastomer balloon at position (*L* > 0, *S = *0, $\theta = 0$) on the passive 2D elastomer membrane (Fig. 2A). Upon supplied volume *V* of the compressed air (see Fig. S4 for the description of the volume-based control strategy), the MAIPAM generated an elongated displacement $\Delta W$ (Fig. 2B). We investigated the influence of *L* on $\Delta W$ by characterizing the static responses of the elongated MAIPAMs under different *V* (see Fig. S5 for the details of the measuring setup). In Fig. 2C, the measured $\Delta W$ is plotted as a function of *V*, with results demonstrating that $\Delta W$ of the MAIPAMs with different *L* (such as 20 mm, 40 mm and 60 mm in Fig. 2C) increases linearly with the increase of *V*. In particular, these curves show similar slopes because of our volume-based control strategy. We can also observe that the maximum value of $\Delta W$ is proportional to *L* because the maximum *V* depends on *L*. Further, we investigated the responses of the elongated MAIPAMs under multiple-cycling actuations (Fig. S6A), which demonstrate that there is a complex hysteresis phenomenon between $\Delta W$ and *V* (Fig. S6B). This is mainly caused by the inherent viscoelastic nonlinearity of the used materials [32–34]. In addition, we characterized the payload capability of the elongated MAIPAMs by measuring their static responses under different payloads (such as 0, 100, 200 and 300 g). The experimental results (Fig. S7) show that with an increase in the payload, the slopes of the $V - \Delta W$ curves remain almost constant while the initial $\Delta W$ is increasing because of the deformation under the payload, which demonstrates a good payload capability of the elongated MAIPAMs.

#### Bending

To form an eccentric elastomer balloon (type II) for the bending actuation, $\theta $ was set as zero, and *S* was the primary design parameter. Without loss of generality, we designed the bending MAIPAMs by assembling the active elastomer balloon at position (*L* = 50 mm, *S > 0*, $\theta = 0$) on the passive 2D elastomer membrane (Fig. 2D). Upon supplied volume *V* of the compressed air, the MAIPAM generates a bending angle $\gamma $ (Fig. 2E). We analyzed the influence of *S* on $\gamma $ by measuring static responses of the bending MAIPAMs under different *V*. The measured $\gamma $ is plotted as a function of *V* in Fig. 2F (see Fig. S8 for the details of the measuring setup). The results demonstrate that when *S* is within a range of 20 to 60 mm, $\gamma $ rapidly increases with the increase of *V*. In addition, with the increase of *S*, the slope of the curves becomes larger because larger *S* can generate a larger bending moment. The payload capabilities of the bending MAIPAMs under different payloads (such as 0, 100, 200 and 300 g) are shown in Fig. S9. We can see that $\gamma $ rapidly decreases with increasing payload, mainly caused by the low stiffness of the used materials.

#### Spiraling

To form a helical elastomer balloon (type III) for the spiraling actuation, the design parameter mainly relies on $\theta $. Thus, we designed the spiraling MAIPAMs by assembling the active elastomer balloon at position (*L *= 40/cos$\theta $ mm, *S* = 20 mm, $\theta $* > 0*) on the passive 2D elastomer membrane (Fig. 2G). Upon supplied volume *V* of the compressed air, the spiraling MAIPAMs generate complex 3D deformations (Fig. 2H). We investigated the influence of $\theta $ on the performances of the spiraling MAIPAMs by measuring the helical deformation under the same *V* (see Fig. S10 for the details of the measuring setup). Figure 2I shows one example of the measured 3D deformation of the spiraling MAIPAMs when *V* equals 120 mL with a pressure of 46.5 kPa. The results indicate that the thread pitch decreases with the increase of $\theta $ within 30° to 60°.

### MAIPAMs with multiple-mode actuations

We next demonstrated that our MAIPAMs are scalable to produce parallel or cascaded multiple-mode actuations with previous design and fabrication approach.

#### Parallel multiple-mode actuation

**Figure 3.**
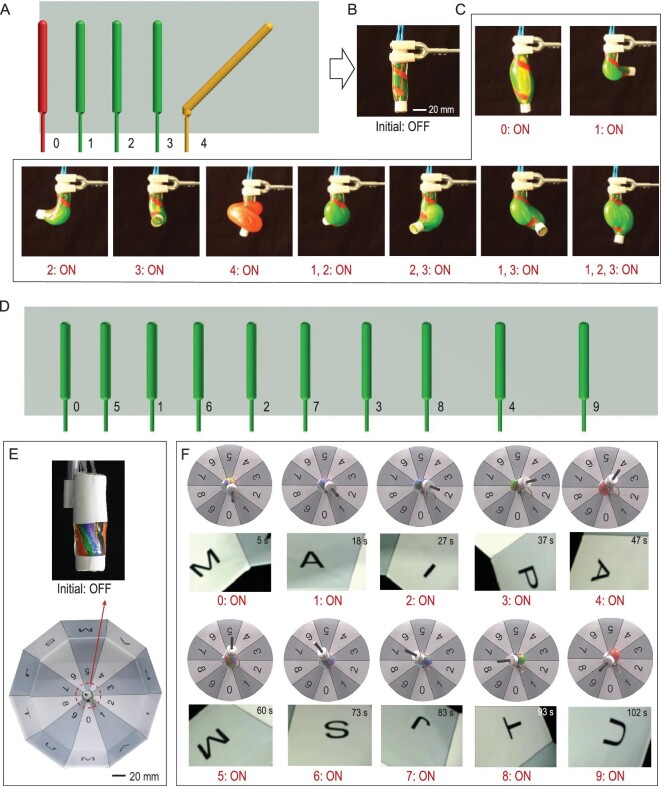
MAIPAMs with parallel multiple-mode actuations. (A) Schematic of the 2D-based design pattern of a MAIPAM with five parallel active elastomer balloons (including one type I balloon of number 0, three type II balloons of numbers 1 to 3 and one type III balloon of number 4). (B) A photograph of the accomplished MAIPAM. (C) By individually actuating the active 3D elastomer-balloon arrays in the order of 0 to 4, the MAIPAM can generate elongation, three bending and spiraling modes, respectively. By simultaneously actuating two active elastomer balloons, 1 and 2, 2 and 3, 1 and 3, the MAIPAM can generate another three bending modes. Further, when active elastomer balloons 1 to 3 are simultaneously actuated, the MAIPAM can achieve an elongation mode because of its symmetric structure (Movie S2). (D) Schematic of the 2D-based design pattern of a MAIPAM with 10 parallel active elastomer balloons (10 type II balloons of numbers 0 to 9) for omnidirectionally bending. (E) A photograph of the MAIPAM assembled into a decagonal prism. (F) The application of the MAIPAM with parallel multiple-mode actuations to record videos in a confined space with a small camera, such as recording the alphabets (‘MAIPAM’ and ‘SJTU’) on 10 surfaces of the decagonal prism (Movie S3).

Parallel multiple-mode actuation of the MAIPAMs was achieved by assembling a number of parallel active elastomer balloons. As an example, Fig. 3A shows a 2D-based design pattern and assembly of a MAIPAM with five parallel active elastomer balloons (including one type I balloon of number 0, three type II balloons of numbers 1 to 3, and one type III balloon of number 4; Fig. S11A). By individually actuating the active 3D elastomer-balloon array in the order of 0 to 4, the MAIPAM (Fig. 3B) generated elongation, and three bending and spiraling modes, respectively, achieving parallel multiple-mode actuations (Fig. 3C and Movie S2). Further, when we simultaneously actuated multiple elastomer balloons, the MAIPAM generated more complex actuations. For example, when the balloons 1 and 2, 2 and 3, 1 and 3 were actuated, the MAIPAM achieved another three bending modes. In addition, when three type II balloons (1 to 3) were actuated at the same time, the MAIPAM generated an elongation mode because of its symmetric actuation.

Next, we demonstrated that the number of the active elastomer balloons in MAIPAMs can be further increased to 10. Figure 3D shows a 2D-based design pattern and assembly of a MAIPAM with 10 parallel active elastomer balloons (10 type II balloons of numbers 0 to 9; Fig. S11B). By selectively actuating the active 3D elastomer-balloon arrays, the MAIPAM could be bent along 10 different directions (Fig. 3E), which can be used to record videos in confined spaces by carrying a small camera, such as recording the alphabets (‘MAIPAM’ and ‘SJTU’) written on 10 surfaces of a decagonal prism (Fig. 3F and Movie S3).

#### Cascaded multiple-mode actuation

**Figure 4.**
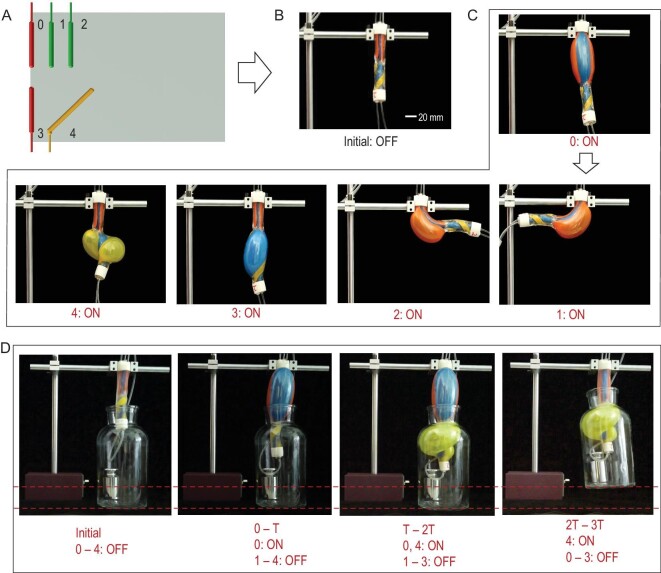
MAIPAMs with cascaded multiple-mode actuations. (A) Schematic of the 2D-based design pattern of a MAIPAM with two cascaded active elastomer-balloon arrays. Array one contains three active elastomer balloons (one type I balloon of number 0 and two type II balloons of numbers 1 and 2) and array two consists of two active elastomer balloons (one type I balloon of number 3 and one type III balloon of number 4). (B) A photograph of the accomplished MAIPAM. (C) Photographs of the cascaded multiple-mode actuations of the MAIPAM. By actuating the active 3D elastomer-balloon arrays in the order of 0 to 4, the MAIPAM can generate elongation, left bending, right bending, elongation and spiraling, respectively (Movie S4). (D) The application of the MAIPAM with cascaded multiple-mode actuations for gripping a glass bottle of weight 600 g (Fig. S12 and Movie S5).

Further, we demonstrated that MAIPAMs can also achieve cascaded multiple-mode actuations by assembling the active elastomer-balloon arrays in series. As an example, Fig. 4A shows the 2D-based design pattern and assembly of a MAIPAM (Fig. S11C), which contains two cascaded active elastomer-balloon arrays, where array one contains three active elastomer balloons (including one type I balloon of number 0 and two type II balloons of numbers 1 and 2) and array two consists of two active elastomer balloons (including one type I balloon of number 3 and one type III balloon of number 4). By actuating the active 3D elastomer-balloon arrays in the order of 0 to 4, the MAIPAM (Fig. 4B) generated elongation, left bending, right bending, elongation and spiraling modes, respectively, therefore achieving both parallel and cascaded multiple-mode actuations (Fig. 4C and Movie S4). In addition, by cooperatively actuating the active 3D elastomer-balloon arrays, the MAIPAM was used to grip a glass bottle with a weight of 600 g (Fig. 4D, Fig. S12 and Movie S5).

### MAIPAMs with multiple materials

We further demonstrated that with our planar design and one-step rolling fabrication approach, the MAIPAMs can conveniently integrate different functional materials into the passive 2D elastomer membrane, resulting in new actuating functions and self-sensing ability.

#### Non-stretchable materials for contraction and twisting

**Figure 5.**
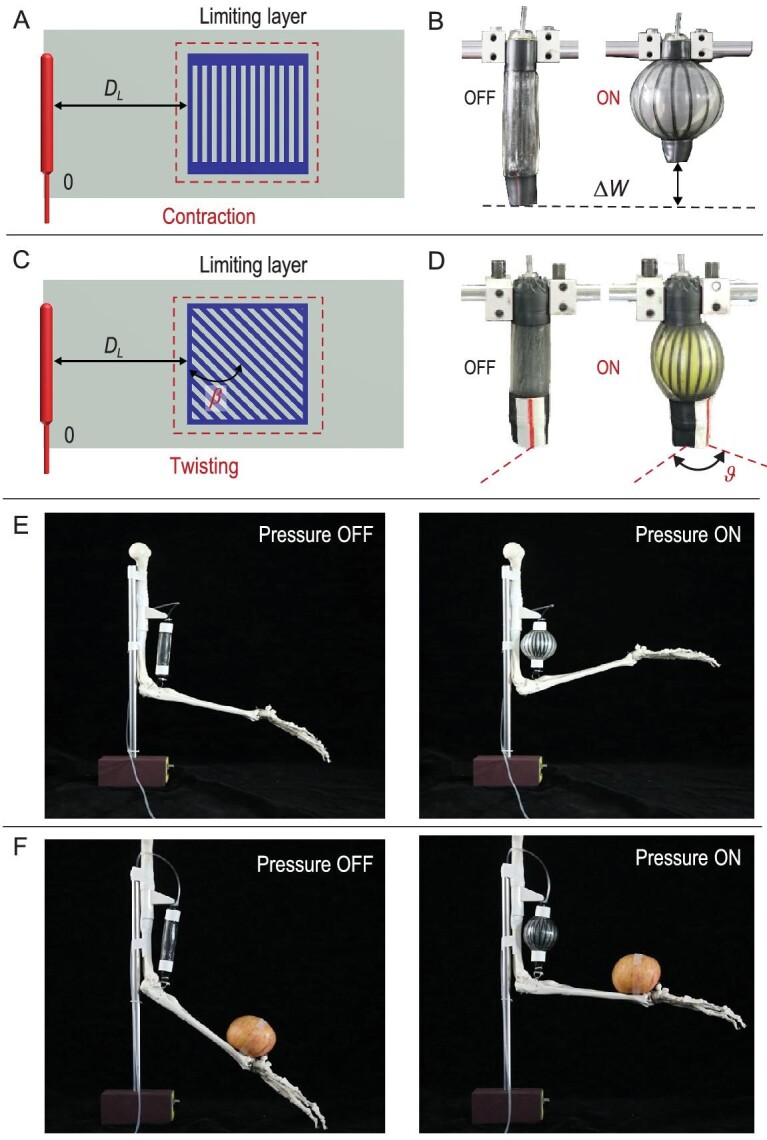
The MAIPAM with non-stretchable materials for contraction and twisting actuation modes. (A) Schematic of the 2D-based design pattern of the contracted MAIPAMs. Based on the elongated MAIPAM (Fig. 2A), a contracted MAIPAM is designed by assembling a non-stretchable laser cutting layer in the passive 2D elastomer membrane. (B) The working principle of the contracted MAIPAMs: upon compressed air, it can generate a contracted displacement $\Delta W$. (C) The 2D-based design pattern and assembly of the twisting MAIPAMs, achieved by tuning the angle $\beta $ of the limiting layer in (A). (D) The working principle of the twisting MAIPAMs: upon compressed air, it can generate a twisting angle $\vartheta $. (E) Application of the contracted MAIPMA for driving a robotic arm (Movie S6). (F) Photographs of the robotic arm lifting an apple of weight 200 g (Movie S6).

Inspired by the fiber-reinforced pneumatic elastomer actuators, we designed contracted MAIPAMs by introducing a 2D limiting layer made of laser cutting non-stretchable cloth (TPU-420D knitted fabric, a thickness of 0.1 mm) into the passive 2D elastomer membrane (Fig. 5A). Under supplied pressure, elongation of the active elastomer balloon was constrained, and the pressure was perpendicular to the limiting layer, generating a contracted actuation (Fig. 5B). Furthermore, we can tune the angle $\beta $ of the limiting layer (Fig. 5C) to achieve twisting actuation for MAIPAMs (Fig. 5D). We employed the contracted MAIPAM to drive a robotic arm for a bending movement (Fig. 5E and Movie S6) and lifting an apple of weight 200 g (Fig. 5F and Movie S6).

#### Compliant hydrogel electrode for self-sensing ability

Next, we demonstrated that our MAIPAMs can achieve a self-sensing ability by integrating a sensor module. Based on the elongated MAIPAM (Fig. 2A), we further assembled a hydrogel-based compliant electrode [35] in the passive 2D elastomer membrane (Fig. S13A). Upon a supplied

pressure, elongation $\Delta W$ of the MAIPAM led to an increase in the electrode's resistance *R*, enabling the MAIPAM to achieve a self-sensing ability by measuring the change of *R*. We investigated the

relationship between *R* and $\Delta W$ by measuring the static responses of the MAIPAM, and *R* is plotted as a function of $\Delta W$ (Fig. S13B). The experimental results demonstrated a linear relationship between *R* and $\Delta W$, which can be expressed as:

(1) \begin{equation*}R = a\Delta W + b,\end{equation*}

where parameters *a* and *b* are fitted as 0.158 and 1.619, respectively. With the self-sensing ability, we developed a closed-loop controller for the MAIPAM to achieve accurate position control and manipulation (see Fig. S14 for the description of the control system). The results demonstrated that the MAIPAM with the position controller can accurately control movement of an egg (Fig. S13C and Movie S7). In contrast, the MAIPAM will push the egg off a cliff without the controller (Fig. S13D and Movie S7). We mainly used this experiment to show the scalable ability and function in integrating multiple materials for self-sensing without considering the hysteresis nonlinearity of the hydrogel sensors [36–38], which is also important for further applications.

### MAIPAMs for soft robotic applications

**Figure 6.**
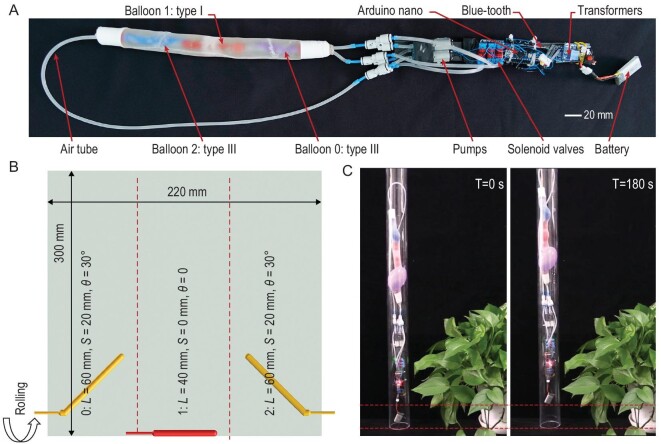
An untethered pipe-climbing robot with a multiple-mode MAIPAM. (A) The structure of the untethered pipe-climbing robot, consisting of a MAIPAM and an untethered control system. (B) Schematic of the 2D-based design pattern of the MAIPAM. The MAIPAM contains three cascaded active elastomer balloons (two type III balloons of numbers 0 and 2, one type I balloon of number 1). Upon compressed air, balloon 1 can generate a periodical elongation-contraction motion while balloons 0 and 2 can form helical shapes that can be used to generate controllable friction forces. By synthetically controlling the periodical motion and the friction forces (Fig. S15), the robot can achieve stable climbing in a pipe-line. (C) Photographs of climbing processes of the untethered pipe-climbing robot in a pipe-line of diameter 55 mm (Movie S8).

Lastly, we demonstrated that the MAIPAMs with multiple-mode actuations can be used directly to develop an untethered pipe-climbing robot (Fig. 6A, see Fig. S15A for the details of the untethered control system). This MAIPAM contains three cascaded active elastomer balloons (two type III balloons of numbers 0 and 2, one type I balloon of number 1; Fig. 6B). Upon a supplied pressure, balloon 1 generated a periodical elongation-contraction movement while balloons 0 and 2 formed helical shapes that can produce controllable friction forces by squeezing the pipe-line. By synthetically controlling the periodical movement and the friction forces (see Fig. S15B for details about the control strategy), the pipe-climbing robot can achieve stable climbing in a pipe-line of diameter 55 mm (Fig. 6C and Movie S8). Differing from reported crawling or climbing soft robots [10,15,39], which generally need to assemble different components (such as actuators and feet), our pipe-climbing robot consists of a single MAIPAM without further assembly process.

## DISCUSSION

In summary, we present a new class of MAIPAMs that consist of active 3D elastomer-balloon arrays reinforced by a passive 2D elastomer membrane. By selectively actuating the active elastomer-balloon arrays, our MAIPAMs show versatile abilities on multiple-mode actuations (including elongation, bending, spiraling, contraction, twisting and their combinations). Using a planar design and one-step rolling fabrication approach, the MAIPAMs are scalable to integrate multiple materials for new actuations and self-sensing functions, such as the limiting layers (non-stretchable materials) for contraction and twisting actuation modes, and compliant electrodes (hydrogel) for self-sensing. With the multiple-mode actuations, the MAIPAMs demonstrate various applications for soft robotic systems, such as carrying a camera for recording videos, gripping or manipulating objects, and climbing a pipe-line.

Our planar design and one-step rolling fabrication approach provides a mold-free yet low-cost method to design and fabricate soft-flexible-rigid coupled soft robots. Although no binding material is needed in this work because of the inherent adhesion of the VHB membrane, MAIPAMs can still be made of non-stick elastomer membranes (such as silicone membranes). The main difference is that additional adhesive material is needed to coat the elastomer membrane before the rolling fabrication.

Based on the design parameters (*L*, *S*, $\theta $, *P*, *W*, *t*), our geometric model (see supplementary text) can directly calculate the position of each active elastomer balloon in the MAIPAMs. The geometric model can also be used to calculate the designed parameters (*L*, *S*, $\theta $, *P*, *W*, *t*) based on the desired actuation modes (see Table S1). We should mention that because of the material nonlinearity (such as viscoelastic nonlinearity), complex deformation and coupling effect, there are still huge challenges to develop an accurate physical model for predicting specific deformation of the MAIPAMs under compressed air. In our future work, we will focus on modeling and compensating those nonlinearities of the MAIPAMs.

## METHODS

### Materials and fabrication

The materials for fabricating MAIPAMs are commercially available products, including the active elastomer balloon (made of latex, Beijing Qing Wei Jia Si Company), the passive elastomer membrane (VHB 4910, a thickness of 1 mm, 3M Company) and the limiting layer (TPU-420D knitted fabric, a thickness of 0.1 mm, Jiaxing Inch Eco-Materials). The fabrication processes of the MAIPAMs based on the planar design and one-step rolling fabrication approach are detailed in Fig. 1 and Fig. S2.

The hydrogel-based compliant electrode for the self-sensing ability is made of polyacrylamide (PAAm) hydrogel containing lithium chloride (LiCl) [35]. The fabrication processes of the electrode are described in Fig. S14A.

### Descriptions of the control platform

As the active elastomer balloons are made of hyper-elastic materials (see Fig. S16 for details of the static responses of the active elastomer balloons), we developed a volume-based control system to actuate MAIPAMs for performance evaluations. The structure and strategy of the volume-based control system are shown in Fig. S4. A barometer (BOOST.CPM.0103, 0–250 kPa) was used to measure the air pressure. For the elongated and bending MAIPAMs, a dSPACE (MicroLabBox DS1202) was adopted to control the volume-based control system. For the spiraling MAIPAMs, a microprogrammed control unit (MCU, STM32F103ZET6) was used to generate control signals for the volume-based control system.

The self-sensing-based closed-loop control system is shown in Fig. S14B, which mainly consists of a LCR meter (Tonghui, TH2838H) for recording the resistance of the electrode, a micro-pump for supplying compressed air and the MCU (STM32F103ZET6) for translating the resistance into the displacement based on $R = a\Delta W + b$ and generating control signals for turning on or off the micro-pump.

For the soft pipe-climbing robot, we developed an untethered control system (Fig. S15) consisting of a lithium battery (3.7 V, 250 mAh), an Arduino Nano (ATMEGA328), two voltage transformers, a blue-tooth, six relays, three solenoid valves and three pumps. The battery was used to supply electrical power for the system. One of the voltage transformers was adopted to generate 5 V for the pumps and blue-tooth, while the other one was used to generate 9 V for the Arduino Nano and the solenoid valves. The blue-tooth was used to receive control signals from the computer. The Arduino Nano was used to generate control signals for the relays and process the blue-tooth signals. Three solenoid valves and three pumps are divided into three groups to control the unloading and loading processes of three active elastomer balloons in the MAIPAM, respectively.

### Data acquisition and processing

We adopted a motion tracking system to capture deformation of the elongated, bending and spiraling MAIPAMs, respectively. The dSPACE (MicroLabBox DS1202) equipped with 16-bit analog-to-digital converters and 16-bit digital-to-analog converters was applied to record the experimental results of the elongated and bending MAIPAMs. The MCU (STM32F103ZET6) equipped with 12-bit analog-to-digital converters and 12-bit digital-to-analog converters was used to record the experimental results of the spiraling MAIPAMs. Matlab software was used to process and visualize the experimental results.

## Supplementary Material

nwab048_Supplemental_Files
