# Supplementary material for: Muscle-fiber array inspired, multiple-mode, pneumatic artificial muscles through planar design and one-step rolling fabrication
Source: Natl Sci Rev. 2021 Mar 24;8(10):nwab048. doi: 10.1093/nsr/nwab048 (PMC8566179; doi:10.1093/nsr/nwab048)
Supplement: nwab048_Supplemental_Files [file nwab048_supplemental_files.zip › Supplementary_data.pdf]

Supplementary information for

Muscle-fiber array inspired, multiple-mode, pneumatic artificial muscles through planar design and one-step rolling fabrication

Jiang Zou[1,2], Miao Feng[1,2], Ningyuan Ding[1,2], Peinan Yan[1,2], Haipeng Xu[1,2], Dezhi Yang[1,2], Nicholas X. Fang[3], Guoying Gu[1,2*], and Xiangyang Zhu[1,2*]

[1]State Key Laboratory of Mechanical System and Vibration, Shanghai Jiao Tong University, Shanghai 200240, China; [2]Institute of Robotics, School of Mechanical Engineering, Shanghai Jiao Tong University, Shanghai 200240, China; [3]Department of Mechanical Engineering, Massachusetts Institute of Technology, Cambridge, MA 02139, USA.

*To whom correspondence should be addressed. Email: guguoying@sjtu.edu.cn; mexyzhu@sjtu.edu.cn.

This file includes:

Text: Geometric model to describe the relationship between the design parameters and the 3D structure of the MAIPAMs.

Table s1. The calculated position of each elastomer balloon in MAIPAMs based on the geometric model.

Fig. s1. Characterization of the stress-strain relationships of the active elastomer balloon and the passive elastomer membrane.

Fig. s2. The manufacturing processes of the MAIPAMs with the planar design and one-step rolling fabrication approach.

Fig. s3. The established cylindrical coordinate for MAIPAMs.

Fig. s4. The volume-based control system for MAIPAMs.

Fig. s5. Experimental setup for evaluating the performance of the elongated MAIPAMs.

Fig. s6. Characterization of the hysteresis effect of the elongated MAIPAMs under cycling tests.

Fig. s7. Characterization of the performance of the elongated MAIPAMs under different payloads.

Fig. s8. Experimental setup for evaluating the performance of the bending MAIPAMs.

Fig. s9. Characterization of the performance of the bending MAIPAMs under different payloads.

Fig. s10. Experimental setup for evaluating the performance of the spiraling MAIPAMs.

Fig. s11. The geometric parameters of the MAIPAMs with multiple-mode actuations.

Fig. s12. The control sequences for gripping processes of the MAIPAM.

Fig. s13. The MAIPAM with a compliant electrode for a self-sensing ability.

Fig. s14. The developed control system for MAIPAMs with the self-sensing ability.

Fig. s15. The untethered control system for the soft pipe-climbing robot.

Fig. s16. Characterization of the static responses of the active elastomer balloon.

Other Supplementary information for this manuscript includes the following:

Movie s1 (.MP4 format) The process of the planar design and one-step rolling fabrication approach for MAIPAMs.

Movie s2 (.MP4 format) The parallel multiple-mode actuations of the MAIPAMs.

Movie s3 (.MP4 format) The application of the parallel multiple-mode actuations for recording videos in confined spaces.

Movie s4 (.MP4 format) The cascaded multiple-mode actuations of the MAIPAMs.

Movie s5 (.MP4 format) The application of the cascaded multiple-mode actuations for gripping.

Movie s6 (.MP4 format) The application of the contracted MAIPAM to drive a robotic arm.

Movie s7 (.MP4 format) The application of the MAIPAM with a self-sensing ability for object manipulation.

Movie s8 (.MP4 format) The application of the MAIPAM for building the untethered soft pipe-climbing robot.

Text: Geometric model to describe the relationship between the design parameters and the 3D structure of the MAIPAMs.

For a MAIPAM, there are six planar design parameters, including the position of the active elastomer balloon (the length *L* of the active elastomer balloon, the distance *S* between the active elastomer balloon and the edge of the passive elastomer membrane, the oblique angle θ) and the geometric parameters of the passive 2D elastomer membrane (the length *P*, the width *W* and the thickness *t*). After the one-step rolling process, the MAIPAM forms a cylindrical shape with active 3D elastomer-balloon arrays. Based on the constant volume of the active elastomer membrane (The volume of active elastomer balloons can be ignorable), the length of the MAIPAM equals *W* and the diameter D_E of the MAIPAM can be expressed as:

$$D_E = \sqrt{\frac{4Pt}{\pi}} \tag{1}$$

For the active elastomer balloon, its initial shape is a cylinder with an outer diameter of 5.0 mm and a wall thickness of 0.3 mm. After the one-step rolling fabrication approach, the active elastomer balloon forms a thin lamina with a length of 7.8 mm and a thickness of 0.6 mm under the squeezing of the passive elastomer membrane. To describe the shape of the passive elastomer membrane in MAIPAMs, we focus on the center line of the active elastomer balloon. Based on the cylindrical coordinate system shown in **fig. s3**, the shape of the passive elastomer membrane can be expressed by a parameterized equation:

$$\begin{aligned} & r = t \times T \\ & \beta = 2\pi \times T \\ & \text{Z=z} \in [0, W] \end{aligned} \tag{2}$$

where (*r*, β, Z) represents the 3D coordinates of the passive elastomer membrane and $T \in [0, T_{\max}]$. Considering the constant length of the passive elastomer membrane, we can obtain:

$$P = \int_0^{T_{\max}} r d\beta = \int_0^{T_{\max}} 2\pi t T dT = \pi t T_{\max}^2 \tag{3}$$

Then,

$$T_{\max} = \sqrt{\frac{P}{\pi t}} \tag{4}$$

Based on (2) and (4), for any active elastomer balloon assembled at the position of (L, S, θ) in the passive elastomer membrane, the corresponding 3D coordinate in the MAIPAM can be written as:

$$
\begin{aligned}
& r_e = t \times T_e \\
& \beta_e = 2\pi \times T_e \\
& Z_e = \begin{cases} \in [0, L] & \theta = 0 \\ \left(\pi t T_e^2 - S\right)\tan\theta & \theta \neq 0 \end{cases} \\
& T_e \in \left[\sqrt{\frac{S}{\pi t}}, \sqrt{\frac{S + L\sin\theta}{\pi t}}\right]
\end{aligned}
\tag{5}
$$

With (2) and (5), we can calculate the position of each active elastomer-balloon in the MAIPAM according to their design parameters, which are listed in **Table s1**.

Table s1. The calculated position of each elastomer balloon in different MAIPAMs based on the geometric model.

	Figure	Balloon label	Balloon type	L/mm	S/mm	θ/rad	r_e /mm	β_e /rad	z_e /mm	T_e
1	Fig. 2A	0	I	20<br>40<br>60	0	0	0	0	[0,20]<br>[0,40]<br>[0,60]	0
2	Fig. 2B	0	II	50	20<br>40<br>60	0	2.52<br>3.57<br>4.37	15.85<br>22.42<br>27.46	[0,50]	2.52<br>3.57<br>4.37
3	Fig. 2C	0	III	46.18<br>56.57<br>80.00	20	$\pi/6$<br>$\pi/4$<br>$\pi/3$	T_e	$2\pi T_e$	$\left(\pi T_e^2 - S\right)\tan\theta$	[2.52, 3.70]<br>[2.52, 4.37]<br>[2.52, 5.33]
4	Fig. 3A	0	I	60	0	0	0	0	[0, 60]	0
		1<br>2<br>3	II	60	80<br>95<br>115	0	5.05<br>5.50<br>6.05	31.71<br>34.55<br>38.01	[0,60]	5.05<br>5.50<br>6.05
		4	III	90	200	$\pi/4$	T_e	$2\pi T_e$	$\left(\pi T_e^2 - S\right)\tan\theta$	[7.98, 9.16]
5	Fig. 3D	0<br>1<br>2<br>3<br>4<br>5<br>6<br>7<br>8<br>9	II	40	78.5<br>116.9<br>162.8<br>216.4<br>277.6<br>95.0<br>136.8<br>186.3<br>243.3<br>307.9	0	5.0<br>6.1<br>7.2<br>8.3<br>9.4<br>5.5<br>6.6<br>7.7<br>8.8<br>9.9	31.41<br>38.33<br>45.23<br>52.15<br>59.06<br>34.55<br>41.46<br>48.38<br>55.29<br>62.20	[0, 40]	5.0<br>6.1<br>7.2<br>8.3<br>9.4<br>5.5<br>6.6<br>7.7<br>8.8<br>9.9
6	Fig. 4A	0<br>3	I	40	0	0	0	0	[0, 40]<br>[60, 100]	0
		1<br>2	II	40	80<br>102	0	5.0<br>5.7	31.70<br>35.80	[0, 40]	5.0<br>5.7
		4	III	57	80	$\pi/4$	T_e	$2\pi T_e$	$100-\left(\pi T_e^2 - S\right)\tan\theta$	[5.05, 6.19]

Note:

i) I, II, and III represent the elastomer-balloon types shown in **Fig. 1B**.

ii) [a, b] means that the corresponding parameter continuously increases from *a* to *b*.

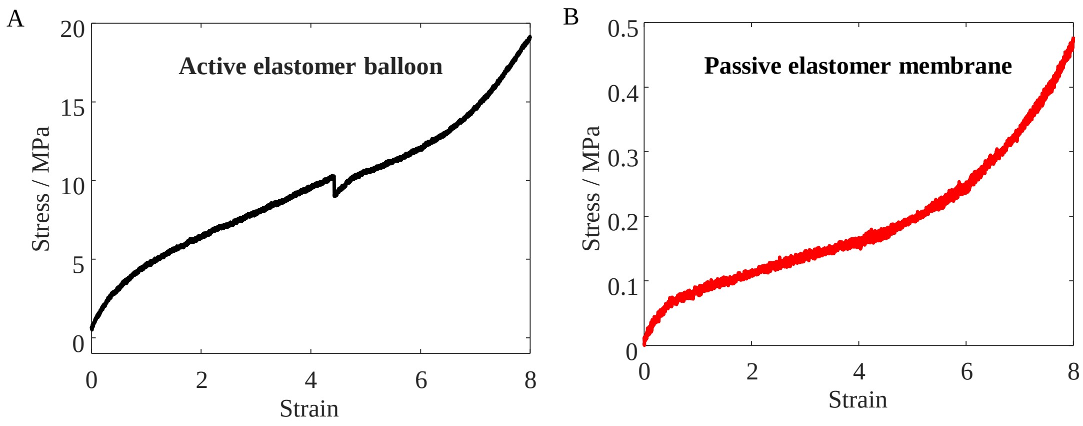

Fig. s1. Characterization of the stress-strain relationships of the active elastomer balloon and the passive elastomer membrane. (A) The stress of the active elastomer balloon is plotted as a function of the applied strain. **(B)** The stress of the passive elastomer membrane is plotted as a function of the applied strain.

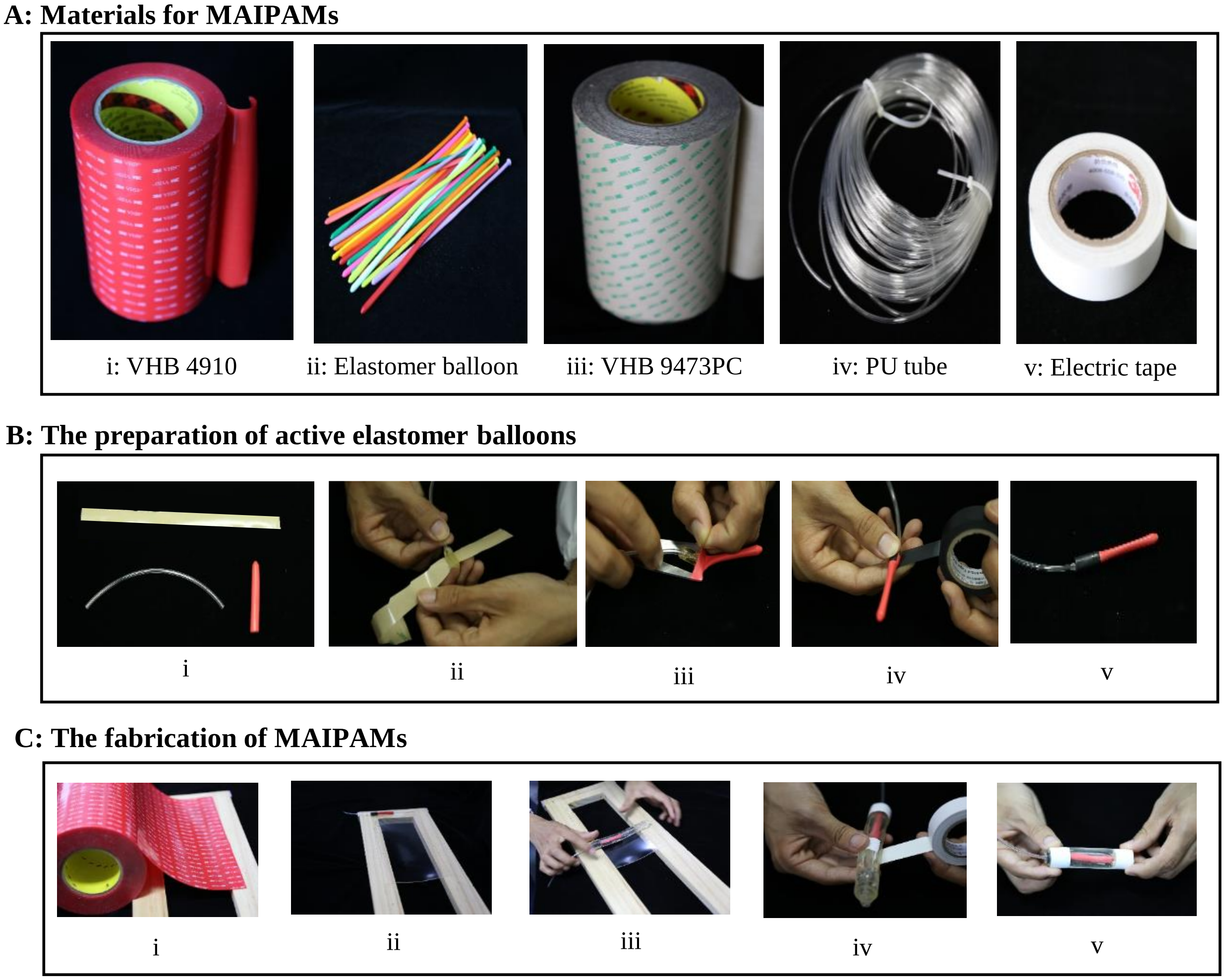

Fig. s2. The manufacturing processes of the MAIPAMs with the planar design and one-step rolling fabrication approach. (A) The materials for fabricating MAIPAMs, including: i) VHB 4910 (3M Company, a thickness of 1 mm) is worked as the passive elastomer membrane; ii) latex elastomer balloons (Beijing Qing Wei Jia Si Company, an outer diameter of 5 mm and a wall thickness of 0.3 mm) are used as the active elastomer balloon; iii) VHB 9473PC (3M Company, a thickness of 0.25 mm) is worked as the adhesion for sealing the interface between air tube and active elastomer balloons; iv) PU tube (NGS, an outer diameter of 3 mm) is utilized for air supply; v) electric tape (3M Company) is used for bundling two ends of accomplished MAIPAMs. **(B)** The preparation of active elastomer balloons involves five steps: i) prepare active elastomer balloons, an air tube and a piece of VHB 4973PC; ii) bundle the air tube with VHB 9473PC for airtightness; iii) install the air tube into the active elastomer balloon; iv) use the electric tape to further seal the active elastomer balloon; v) finish the fabrication of the active elastomer balloons. **(C)** The fabrication of the MAIPAMs involves five steps: i) cut a piece of the VHB 4910 and then adhere it to a supporting frame; ii) assemble the

active elastomer balloons in the passive 2D elastomer membrane; iii) roll the passive elastomer membrane into 3D MAIPAMs; iv) use the electric tape to bundle the MAIPAM for airtightness; v) finish the MAIPAM by removing the residual materials.

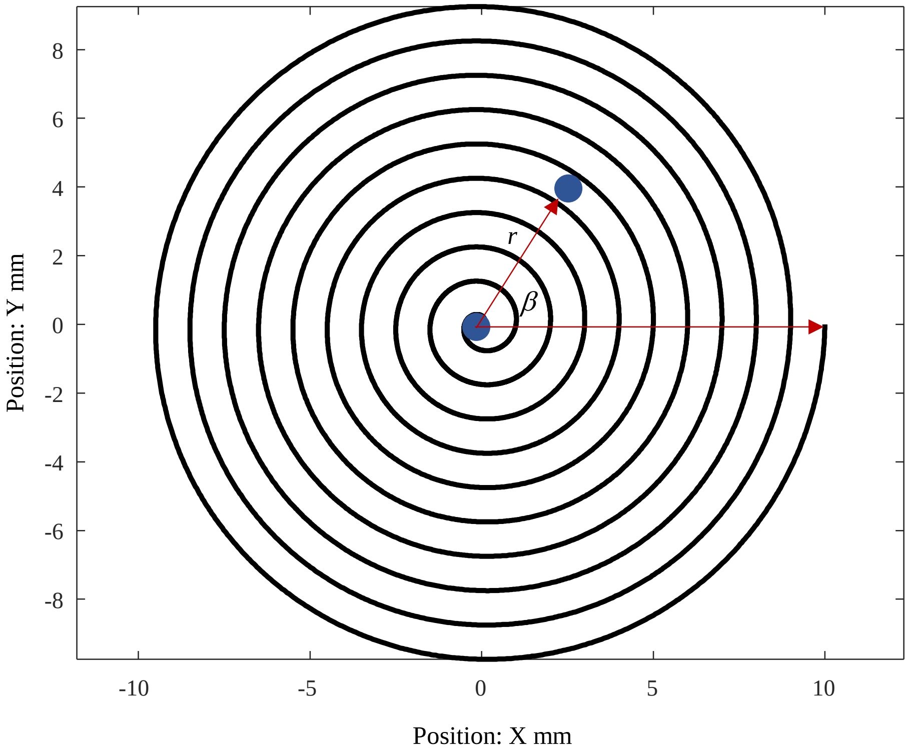

Fig. s3. The established cylindrical coordinate for MAIPAMs. The blue points represent center lines of the active elastomer balloons. The black line is the edge of the passive elastomer membrane after the rolling process. The Z-axis is vertical to the plane.

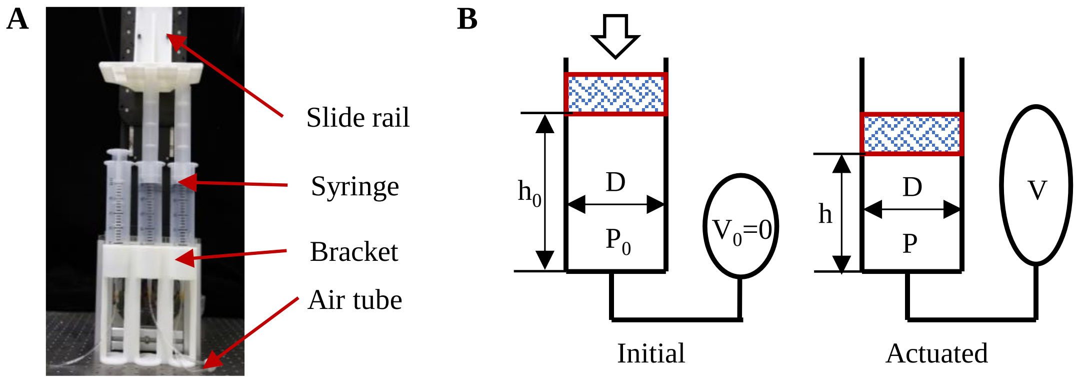

Fig. s4. The volume-based control system for MAIPAMs. (A) The volume-based control system mainly consists of three syringes for air sources, a slide rail (SIGMAKOKI, HST-200) for pushing or pulling the syringes, a bracket for installing those syringes and an air tube for connecting with MAIPAMs. **(B)** The working principle of the volume-based control system. At the initial state, the pressure in each syringe is P_0 (the atmosphere pressure), the height of each syringe equals h_0 and the volume of the air in MAIPAMs can be ignored. When the slide rail pushes the syringes to move a distance $\Delta h = h_0 - h$, the pressure in the syringes and MAIPAMs increases to P and the volume of the compressed air in MAIPAMs is V. Based on the ideal gas state equation, V can be obtained by:

$$V = \frac{n\pi D^2}{4}\left(\frac{P_0 h_0}{P} - h\right) \tag{6}$$

where D represents the diameter of the syringes and n is the number of used syringes. Based on the measured P and applied displacement Δh, we can figure out the volume V of the input air. We should mention that n equals 3 for the experimental setups in **fig. s5** and **fig. s7** while n equals 2 for the experimental setup in **fig. s10**.

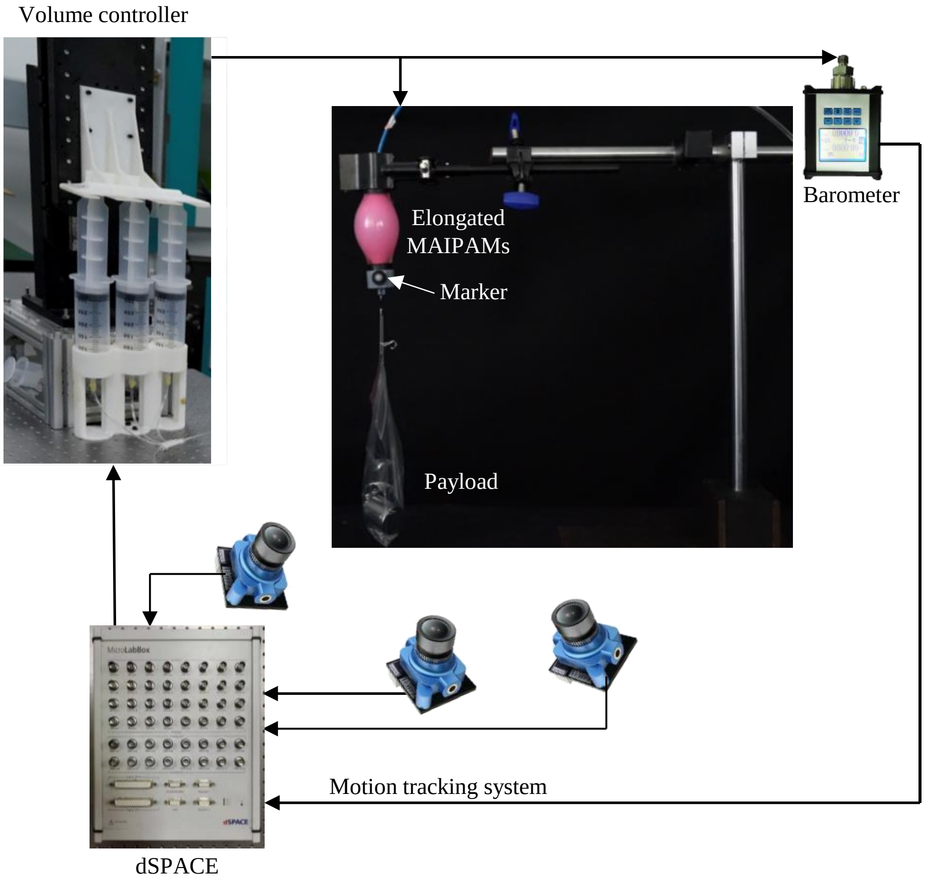

Fig. s5. Experimental setup for evaluating the performance of the elongated MAIPAMs. The volume controller (**fig. s4**) is adopted to supply compressed air. A barometer (BOOST.CPM.0103, 0-250 kPa) is used for measuring the air pressure. A motion tracking system (OptiTrack, Prime 13) is adopted to capture Δw of the elongated MAIPAMs. A dSPACE (MicroLabBox DS1202) is utilized to generate control signals for the volume controller and acquire the air pressure P from the barometer and Δw from the motion tracking system. A constant payload is applied to the elongated MAIPAM. The sampling time is set to be 40 ms.

To measure Δw of the elongated MAIPAMs, we fix one end of the elongated MAIPAMs with a payload on an iron support. The measuring processes involve five steps: i) keep the slide rail and elongated MAIPAMs at the initial state and record the initial position; ii) apply a constant pushing velocity (1 mm/s) to the syringe by the slide rail for loading; iii) record Δw and the corresponding P and applied displacement Δh; iv) when the elongated MAIPAM reaches the maximum elongation, apply a pulling velocity (-1 mm/s) to the syringe by the slide rail for unloading; v) after 6 circles, stop the measurement; vi) change the payload and repeat the above steps.

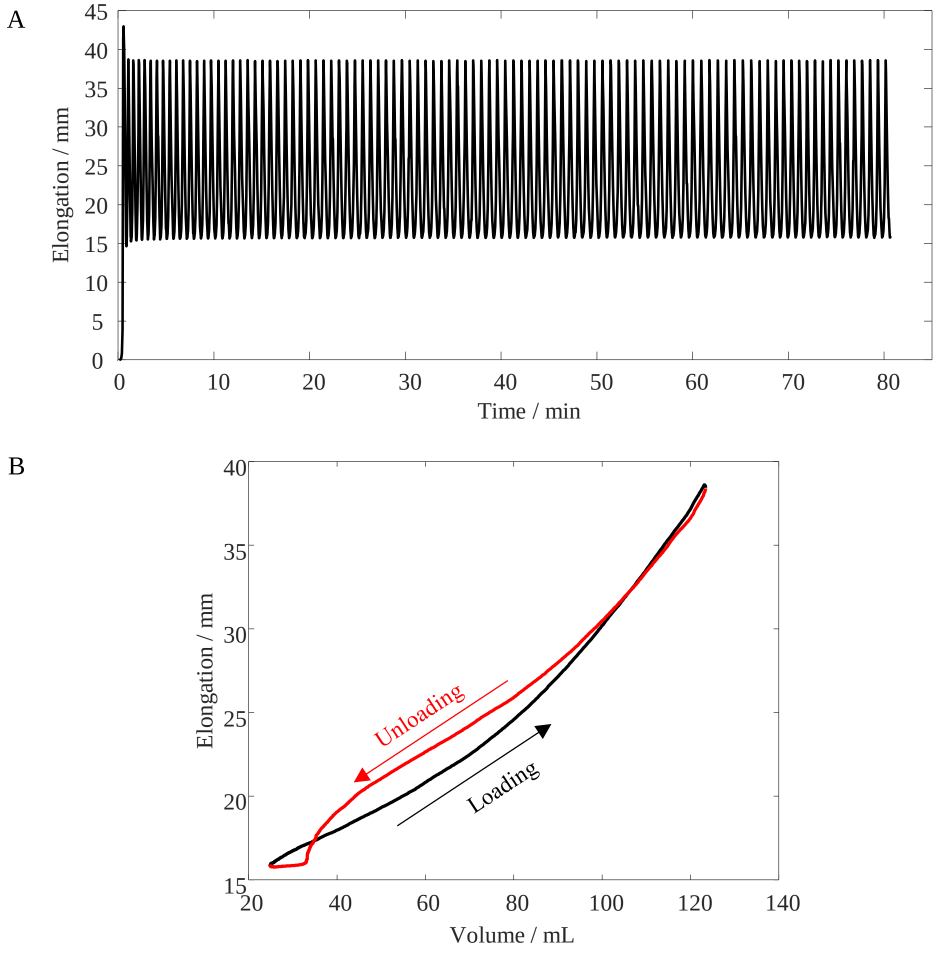

Fig. s6. Characterization of the hysteresis effect of the elongated MAIPAMs under cycling tests. Based on the above experimental setup and measuring process (**fig. s5**), we measure the responses of the elongated MAIPAMs under multiple-cycling actuations with a constant loading velocity (1 mm/s). **(A)** ΔW is plotted as a function of time. **(B)** ΔW is plotted as a function of V at the last cycling test. It can be observed that there is a complex hysteresis phenomenon. We should mention that the hysteresis phenomenon is caused by the viscoelastic nonlinearity of the used materials (both the elastomer balloon and VHB 4910 in this work) [32-34]. In addition, based on the experimental results, we can see that our MAIPAMs can work with multiple-cycling actuations without breakdown or detachment between the passive elastomer membrane and active elastomer balloons.

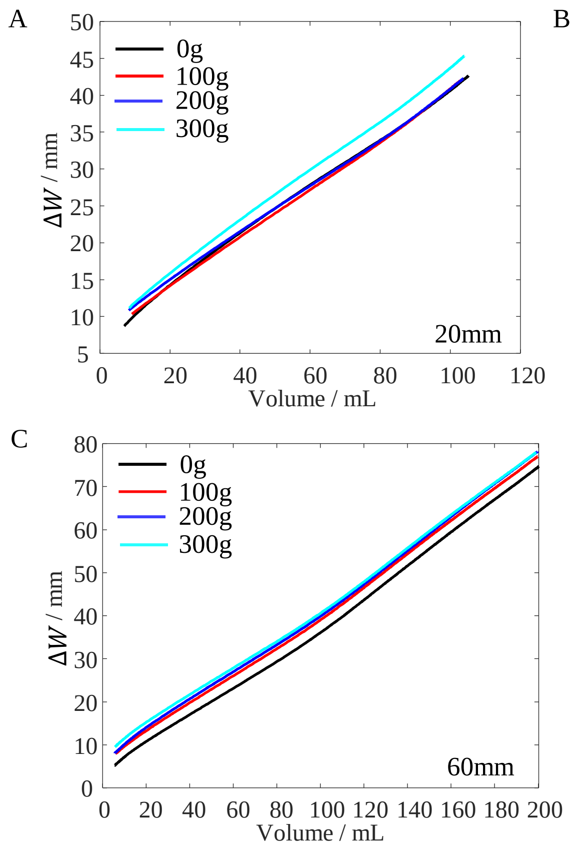

Fig. s7. Characterization of the performance of the elongated MAIPAMs under different payloads. (A), **(B)**, and **(C)** ΔW is plotted as a function of the volume of input air under different payloads (0 g, 100 g, 200 g, and 300 g) when L equals 20 mm, 40 mm, and 60 mm, respectively. We can see that: i) the initial ΔW increases with the increase of the payload, due to the elastic deformation of the elongated MAIPAMs; ii) The slopes of $V-\Delta W$ curves almost are independent on L and the payload. Therefore, the elongated MAIPAMs show a good payload capability along the elongated direction. (Each curve represents average experimental results of five measurements.)

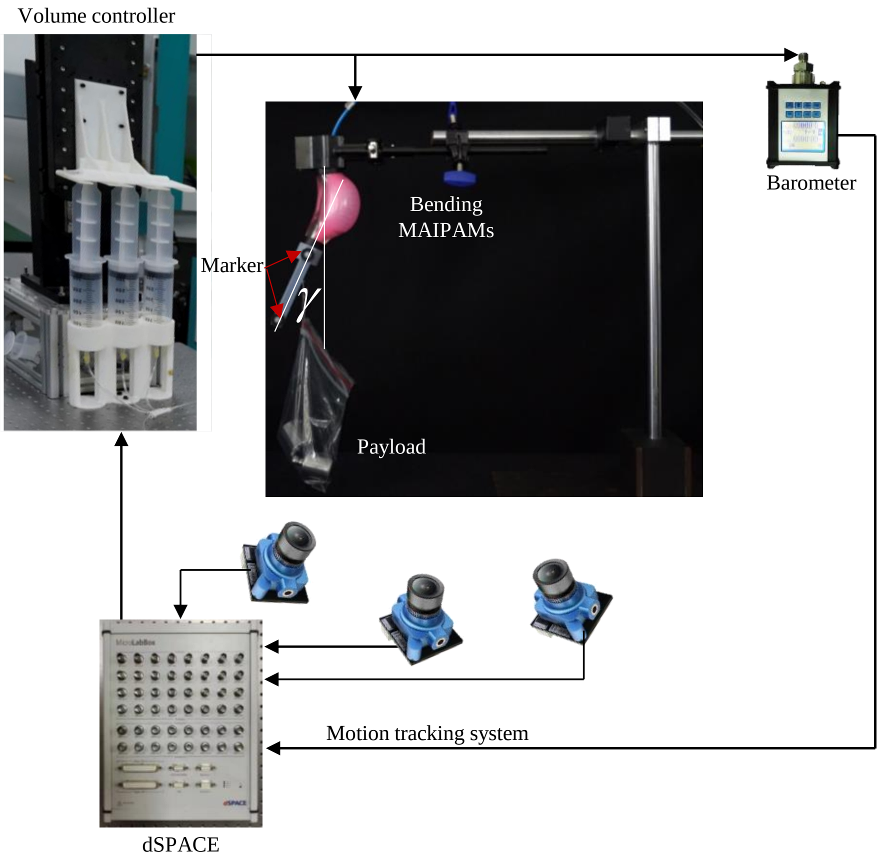

Fig. s8. Experimental setup for evaluating the performance of the bending MAIPAMs. The volume controller (**fig. s4**) is adopted to supply compressed air. A barometer (BOOST.CPM.0103, 0-250 kPa) is used for measuring the air pressure. A motion tracking system (OptiTrack, Prime 13) is adopted to capture the bending angle γ of the bending MAIPAMs. A dSPACE (MicroLabBox DS1202) is utilized to generate control signals for the volume controller and record the air pressure from the barometer and the bending angle from the motion tracking system. A constant payload is applied to the bending MAIPAM. The sampling time is set to be 40 ms.

To measure γ, we fix one end of the bending MAIPAMs on an iron support. The measuring processes involve six steps: i) keep the slide rail and bending MAIPAMs at the initial state and record the initial position; ii) apply a constant pushing velocity (1 mm/s) to the syringe by the slide rail for loading; iii) record γ and the corresponding air pressure P and applied displacement Δh; iv) when the bending MAIPAM reaches the maximum bending angle, apply a reverse velocity to the syringe by the slide rail for unloading; v) after 6 circles, stop the measurement; vi) change the payload and repeat the above steps.

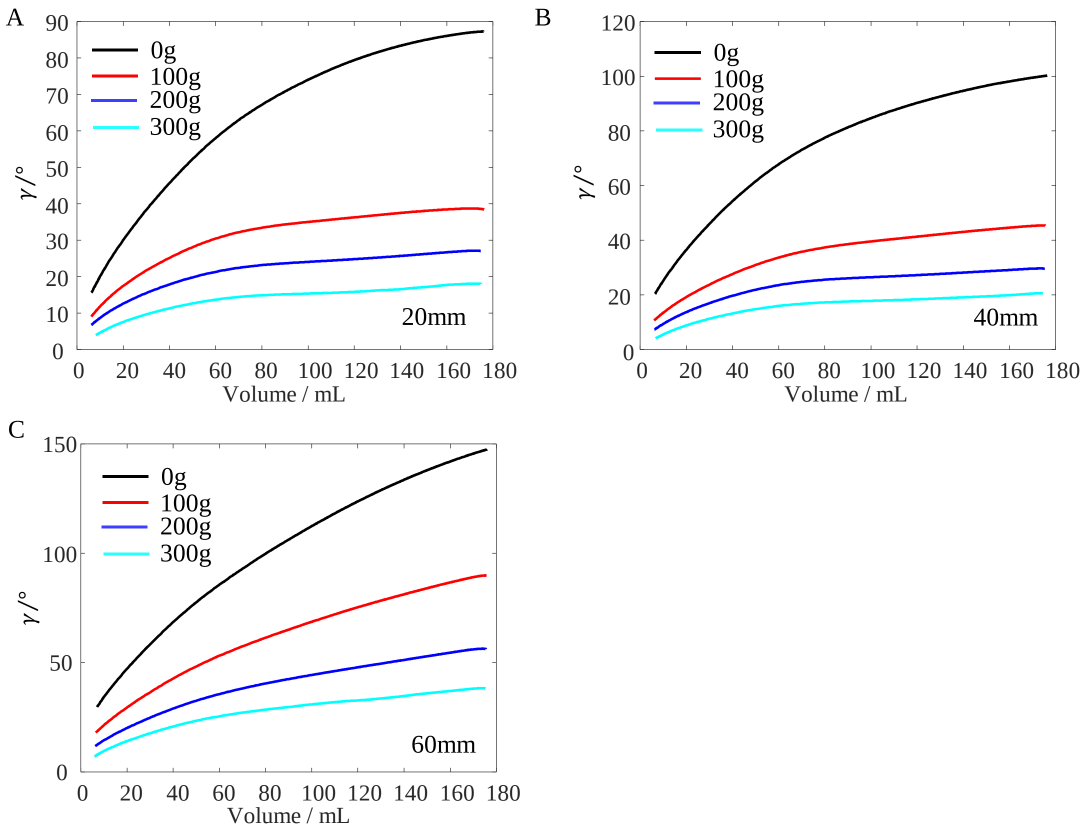

Fig. s9. Characterization of the performance of the bending MAIPAMs under different payloads. (A), **(B)** and **(C)** γ is plotted as a function of V under different payloads (0 g, 100 g, 200 g, and 300 g) when S equals 20 mm, 40 mm, and 60 mm, respectively. (Each curve represents average experimental results of five measurements.)

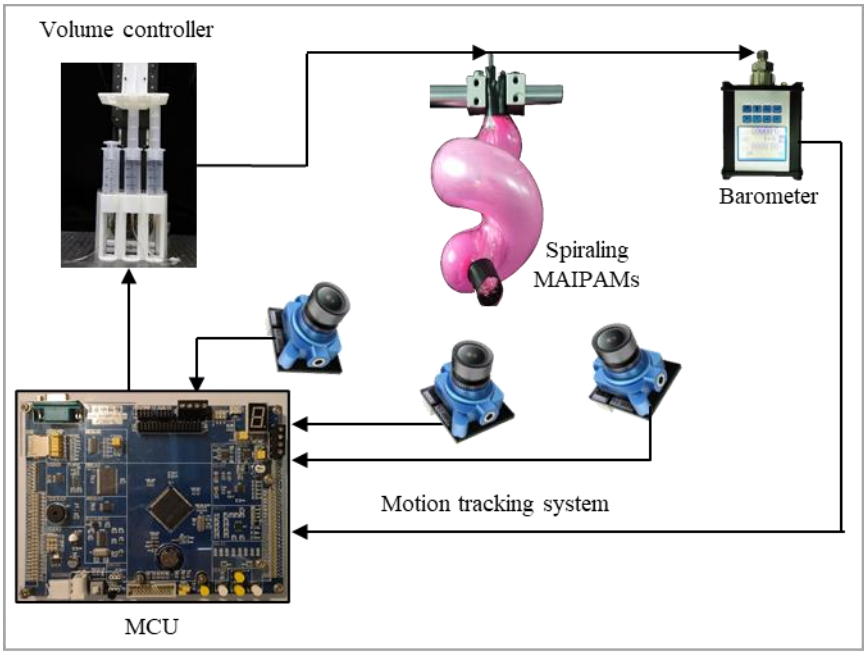

Fig. s10. Experimental setup for evaluating the performance of the spiraling MAIPAMs. The volume controller (**fig. s4**) is adopted to supply compressed air. A barometer (BOOST.CPM.0103, 0-250 kPa) is used for measuring the air pressure. A motion tracking system (OptiTrack, Prime 13) is adopted to capture the 3D spiraling deformation of the spiraling MAIPAMs. A MCU (STM32F103ZET6) is utilized to generate control signals for the volume controller and recording the air pressure from the barometer and the 3D shape from the motion tracking system. The sampling time is set to be 300 ms.

To measure the spiraling deformation of spiraling MAIPAMs, we fix one end of spiraling MAIPAMs on an iron support. The measuring processes involve five steps: i) keep the slide rail and spiraling MAIPAMs at the initial state and record the initial position; ii) apply a displacement Δh to the syringe by the slide rail; iii) wait for some time to let the spiraling MAIPAMs become stable; iv) record the 3D spiraling shape and the corresponding air pressure P *and* V of the input air; v) repeat the measurement by further applying a displacement Δh until the spiraling MAIPAMs reach the maximum deformation.

A

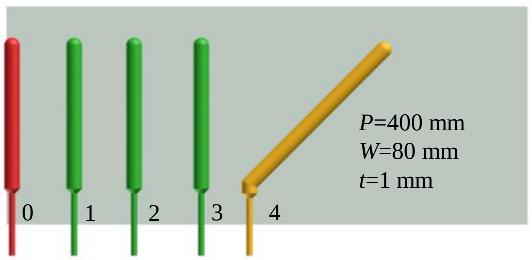

Balloon	0	1	2	3	4
L/mm	60	60	60	60	90
S/mm	0	80	95	115	200
θ/°	0	0	0	0	45°

B

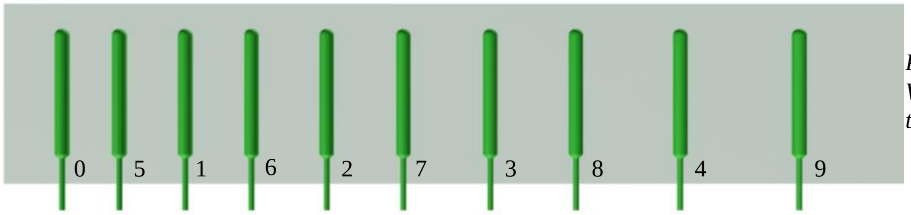

P=706.8 mm
W=60 mm
t=1 mm

Balloon	0	5	1	6	2	7	3	8	4	9
L/mm	40	40	40	40	40	40	40	40	40	40
S/mm	78.5	95.0	116.9	136.8	162.8	186.3	216.4	243.3	277.6	307.9
θ/°	0	0	0	0	0	0	0	0	0	0

C

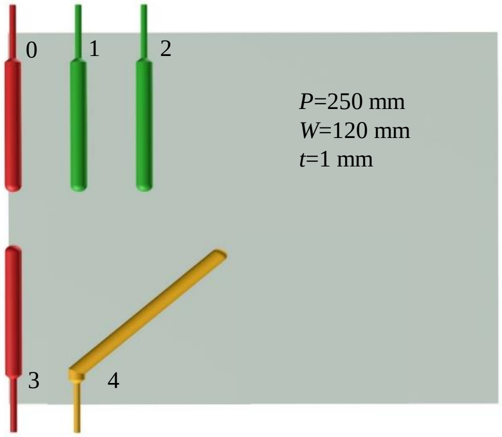

Balloon	0	1	2	3	4
L/mm	40	40	40	40	57
S/mm	0	80	102	0	80
θ/°	0	0	0	0	45°

Fig. s11. The geometric parameters of the MAIPAMs with multiple-mode actuations. (A) The MAIPAM in **Fig. 3A**; **(B)** The MAIPAM in **Fig. 3D**; **(C)** The MAIPAM in **Fig. 4A**.

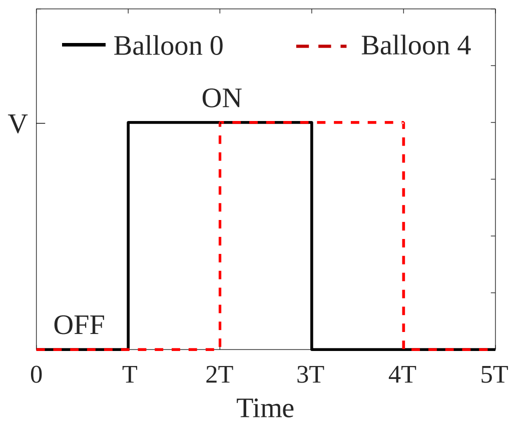

Fig. s12. The control sequences for gripping processes of the MAIPAM. The gripping processes in **Fig. 4D** can be described as:

i) 0~T: At the initial state, the MAIPAM is at the top of the bottle and all the elastomer balloons keep the initial state;

ii) T~2T: Balloon 0 is firstly actuated to generate an elongation that enables the MAIPAM to get into the bottle;

iii) 2T~3T: Balloon 4 is actuated to generate spiraling, resulting in a helical shape to support the bottle;

iv) 3T~4T: The air pressure of the balloon 0 decreases to zero and the contraction of the balloon 0 lifts the bottle;

v) 4T~5T: The air pressure of balloon 4 decreases to zero and MAIPAM recovers its initial state.

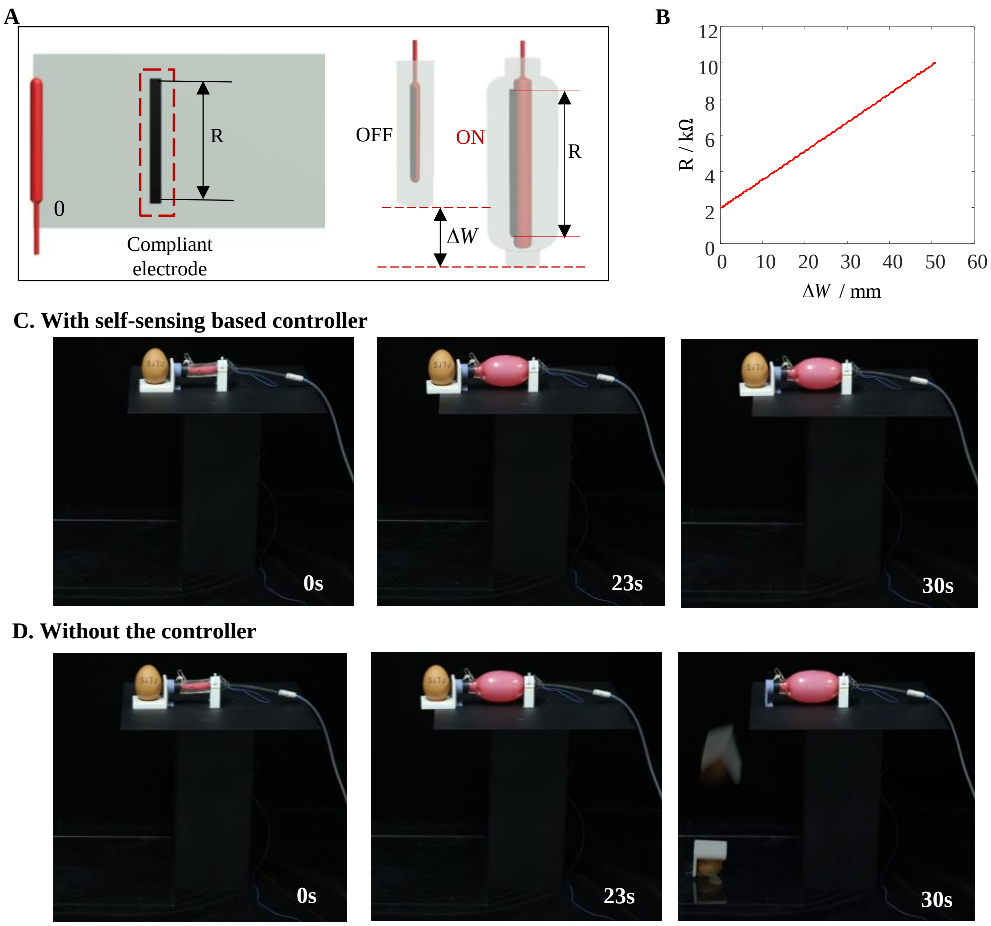

Fig. s13. The MAIPAM with a compliant electrode for a self-sensing ability. **(A)** Schematic of the 2D based design pattern of the MAIPMAs with a self-sensing ability. The self-sensing ability is achieved by assembling a compliant electrode in the passive 2D elastomer membrane of the elongated MAIPAM in **Fig. 2A**. The elongation ΔW of the MAIPAM leads to an increase in the resistance R of the electrode, enabling a self-sensing ability by detecting the change of R. We should mention that due to their inherent viscoelastic nonlinearity of the used soft materials, there will be a hysteresis phenomenon between ΔW and R under periodical actuations [36-38]. In the current work, we mainly aim to show the capability of our fabrication method that can be scalable to integrate multiple materials for additional sensing functions. For simplicity, we only use the sensing data of the loading process of R as the threshold for the feedback control without considering the hysteresis effect of the sensor. **(B)** R is plotted as a function of ΔW. **(C)** The application of the MAIPAM with a self-sensing ability. With a self-sensing based closed-loop controller (**fig. s14**), the MAIPAM can be used to accurately control the position of an egg (**movie s7**). **(D)** The comparison of the MAIPAM without the controller. The MAIPAM pushes the egg to drop off a cliff (**movie s7**).

A

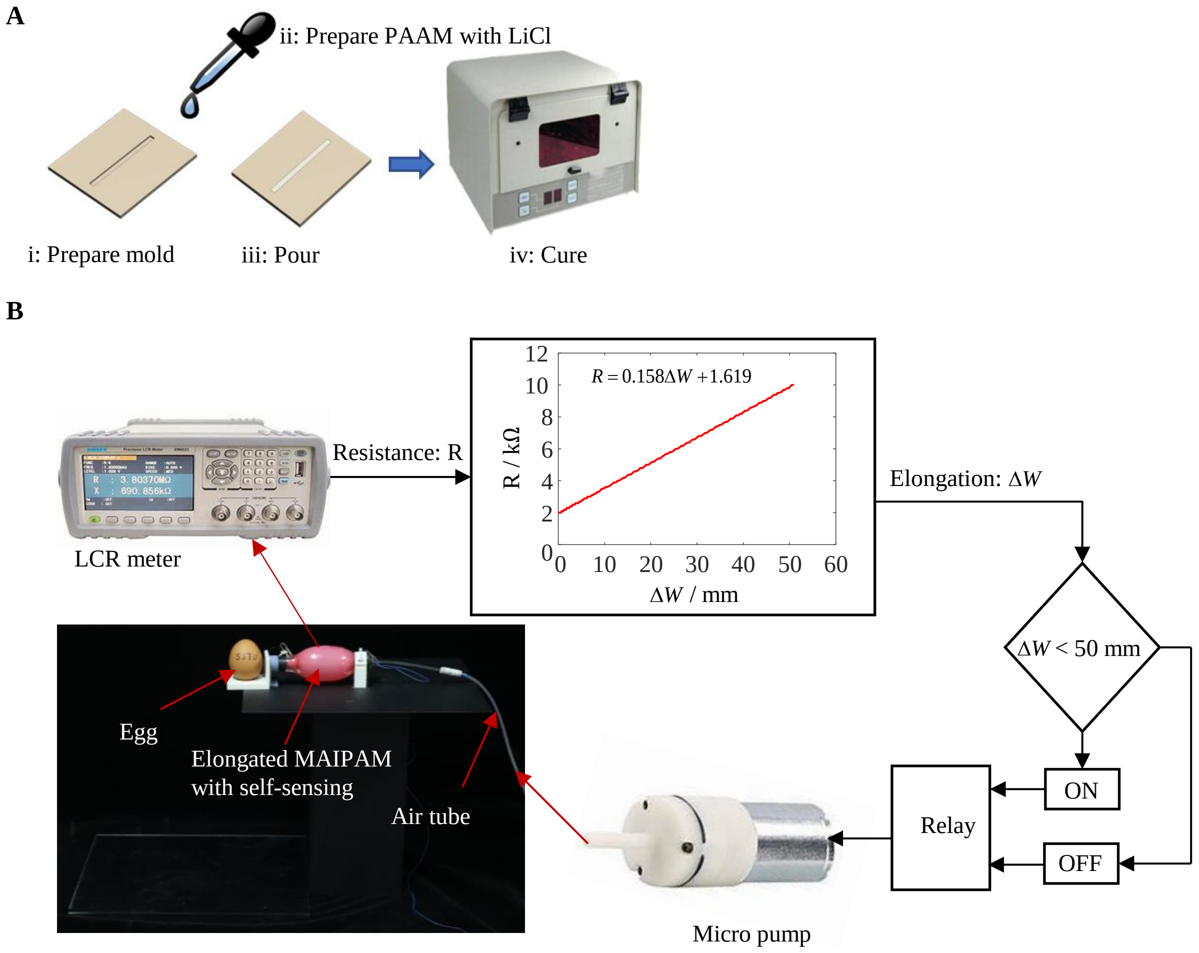

Fig. s14. The developed control system for MAIPAMs with the self-sensing ability. (A) The fabrication of the hydrogel-based electrode involves four steps: i) prepare a mold with a cuboid groove (length 50 mm, width 2 mm, height 1 mm, made of laser cutting VHB 4910); ii) prepare hydrogel solution based on the work proposed in [35]; iii) pour the hydrogel solution into the mold; iv) cure the hydrogel under UV light for 30 minutes. After those processes, we can get a hydrogel-based compliant electrode with a length of 50 mm, a width of 2 mm and a thickness of 1 mm. **(B)** The closed-loop control system with the self-sensing ability. It mainly consists of a LCR meter (Tonghui, TH2838H) for recording the resistance of the electrode, a micro-pump for supplying compressed air and a MCU (STM32F103ZET6) for translating R into the ΔW based on $R = a\Delta W + b$ and generating control signals for turning on or off the micro-pump. The control processes can be described as: i) keep the elongated MAIPAM at the initial state; ii) set the desired displacement to be 50 mm; iii) use the elongated MAIPAM to manipulate an egg. When ΔW is less than 50 mm, the micro-pump keeps working. Once ΔW reaches 50 mm, the micro-pump stops and the position of the egg keeps stable.

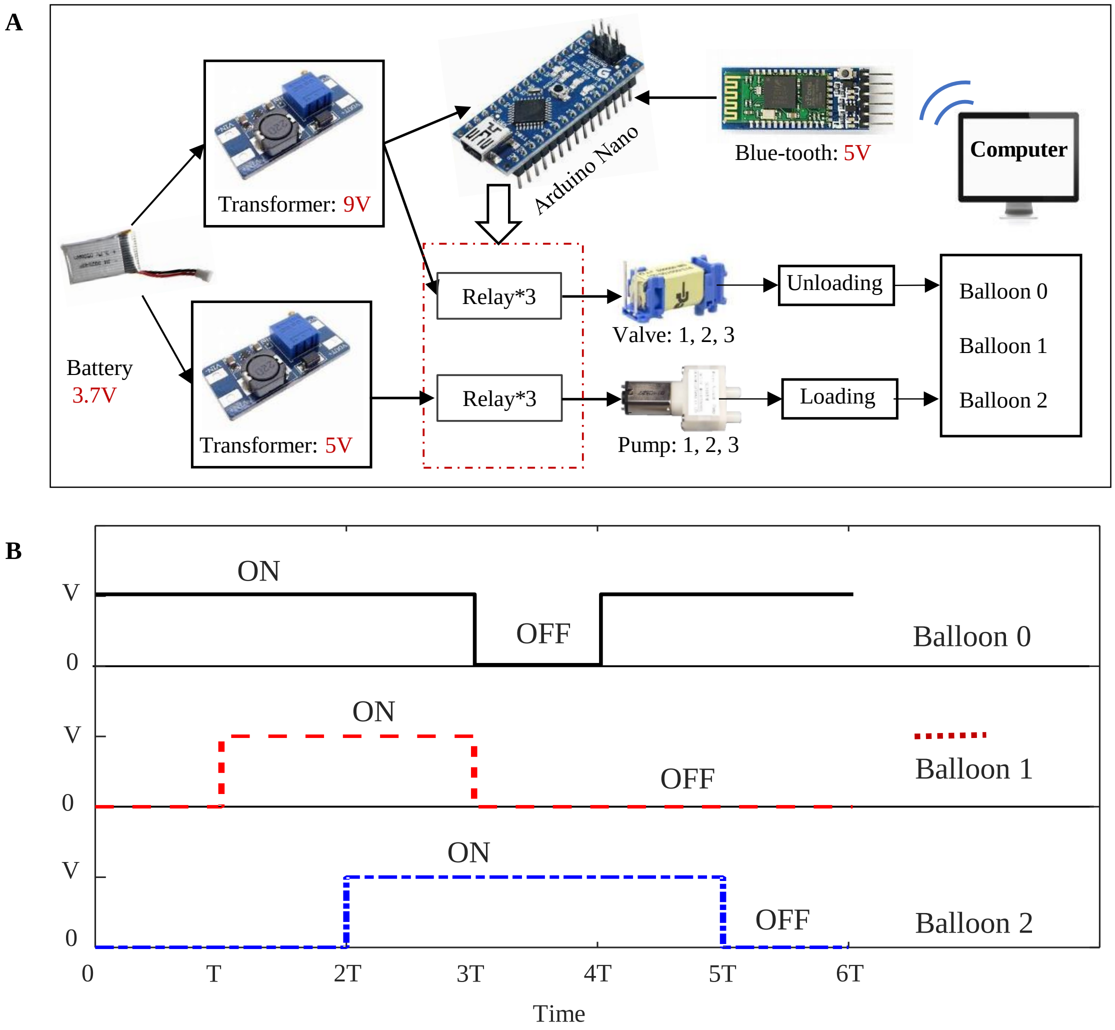

Fig. s15. The untethered control system for the soft pipe-climbing robot. (A) The developed untethered control system. A lithium battery (3.7 V, 250 mAh) is used for supplying electrical power for the system. A voltage transformer is adopted to generate 9 V for the Arduino Nano and solenoid valves while the other one is used to generate 5 V for the pumps and blue-tooth. The blue-tooth is adopted to receive control signals from the computer. The Arduino Nano is utilized to generate control signals for the relays and process the blue-tooth signals. Three solenoid valves and three pumps are divided into three groups to separately control the loading and unloading processes of three active elastomer balloons in the MAIPAM (Valve 1 and pump 1 for the balloon 0, named as end section; Valve 2 and pump 2 for the balloon 1, named as middle section; Valve 3 and pump 3 for the balloon 2, named as head section), respectively. **(B)** The control sequences for climbing involve six steps:

i) 0~T: Balloon 0 is actuated to generate a friction force to support the weight of the robot and prevent from dropping while balloons 1 and 2 keep the initial state.

ii) T~2T: Balloon 1 is actuated to generate elongation that pushes the head section forward, owing to the friction force of the end section is larger than that of the head section.

iii) 2T~3T: Balloon 2 is actuated to generate a friction force.

iv) 3T~4T: The compressed air in the balloons 0 and 1 is released. The friction force of the end section is disappeared while the recovery of balloon 1 pulls the end section forward, because the friction force of the end section is smaller than that of the head section.

v) 4T~5T: Balloon 0 is re-actuated to generate friction force again.

vi) 5T~6T: The compressed air in balloon 2 is released to recover its initial state.

By repeating the above six steps, the pipe-climbing robot can achieve stable climbing in a pipe-line with a diameter of 55 mm. In addition, cost of transport is another important index to evaluate the performance of the pipe-climbing robot and control system, which will be investigated in our future work.

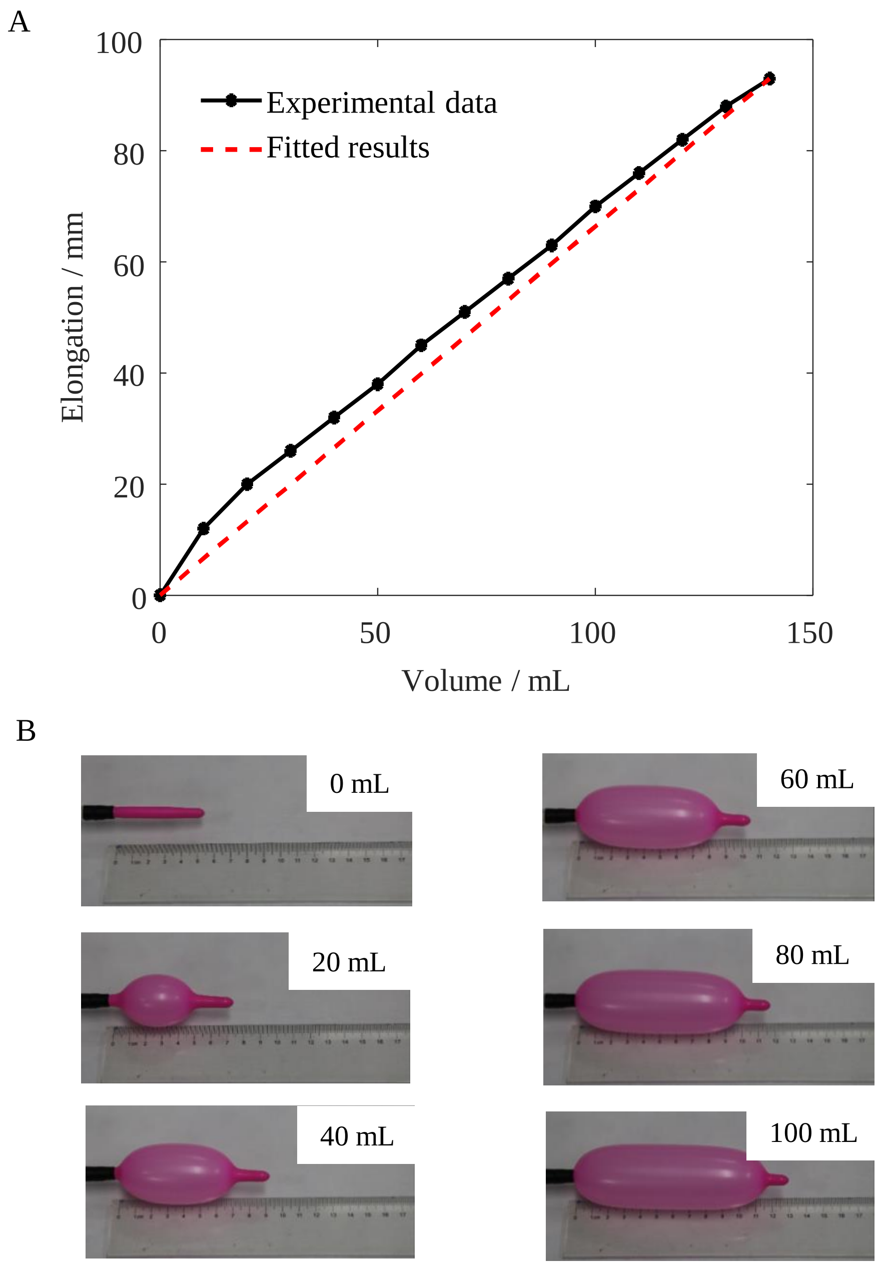

Fig. s16. Characterization of the static responses of the active elastomer balloon. (A) The elongation of the active elastomer balloon is plotted as the function of the input volume *V* of the compressed air. It demonstrates that the elongation proximately linearly increases with the increase of *V*. **(B)** The pictures of the active elastomer balloons under different *V*.

The experimental results also demonstrate that: the deformation of the active elastomer balloon can be divided into three stages: i) when *P* is below a specific value (defined as critical pressure), there is no visible elongation. ii) once *P* reaches the critical pressure, the active elastomer balloon starts to generate a local bulging area (Because the active elastomer balloon is made of hyper-elastic materials) that dominates the

elongation. In the meantime, P almost keeps constant as the development of the bulging area. iii) when the active elastomer balloon is fully bulged, the elongation almost reaches its maximum value. Further inputting compressed air leads to an increase of P and a mechanical breakdown of the active elastomer balloon. It should be noted that we can observe a similar deformation process for different MAIPAMs. Therefore, we develop a volume-based control system for conveniently controlling the MAIPAMs that is detailed in **fig. s4**.

Supplementary Movies

Movie s1 (.MP4 format) The process of the planar design and one-step rolling fabrication approach for MAIPAMs.

Movie s2 (.MP4 format) The parallel multiple-mode actuations of the MAIPAMs.

Movie s3 (.MP4 format) The application of the parallel multiple-mode actuations for recording videos in confined spaces.

Movie s4 (.MP4 format) The cascaded multiple-mode actuations of the MAIPAMs.

Movie s5 (.MP4 format) The application of the cascaded multiple-mode actuations for gripping.

Movie s6 (.MP4 format) The application of the contracted MAIPAM to drive a robotic arm.

Movie s7 (.MP4 format) The application of the MAIPAM with a self-sensing ability for object manipulation.

Movie s8 (.MP4 format) The application of the MAIPAM for building the untethered soft pipe-climbing robot.
